# Lane clearance approach for emergency vehicles in highways network

**DOI:** 10.1371/journal.pone.0276988

**Published:** 2022-11-04

**Authors:** Anshu Khatri, Mathi Senthilkumar

**Affiliations:** Department of Computer Science and Engineering, Amrita School of Computing, Coimbatore, Amrita Vishwa Vidyapeetham, India; Al Mansour University College-Baghdad-Iraq, IRAQ

## Abstract

The steady rise in the number of vehicles in modern urban areas and poor conformance to traffic laws (or rules) lead to avoidable commute delays and traffic jams. Also, there is an increased likelihood of road accidents requiring immediate medical attention. Emergency vehicle (EV) service is adversely affected due to the unavailability of the clear lane that supports EVs to get to their endpoint without being delayed along the national highways. Moreover, the EV’s sirens have a limited range, which may fail to reach and notify other vehicular traffic about its mobility promptly. In addition to this range constraint, EVs in motion often lose their communication links while moving from network to network. This paper delineates an approach using the next generation mobile internet protocol, for automated pathway clearance, for EVs. The prototype model of the proposed approach was experimentally designed and implemented. The proposed design concept is validated by performance characterization via numerical analysis.

## 1. Introduction

Frequent reports of traffic jam conditions have been on an unabated rise by each day in cities of developing countries like India, ensuing in loss of productivity, localized air pollution, and the delay of many important tasks. One of the crucial services that are highly impacted is the EV service, such as ambulance, fire engine, and police patrol. Consequently, local governments have an imperative need to establish direct, fast and efficient transportation by EVs with minimal wait times [1]. Video surveillance, acoustical method and global positioning system (GPS) technologies are primarily employed to track vehicles in intelligent transport systems. Cameras can be placed along the roadsides, in front of buildings, parks and other places to capture, record, and transmit images to the server of the traffic surveillance system for observation and interpretation of traffic conditions, followed by the immediate action of the authority of traffic management system.

**Fig 1:**
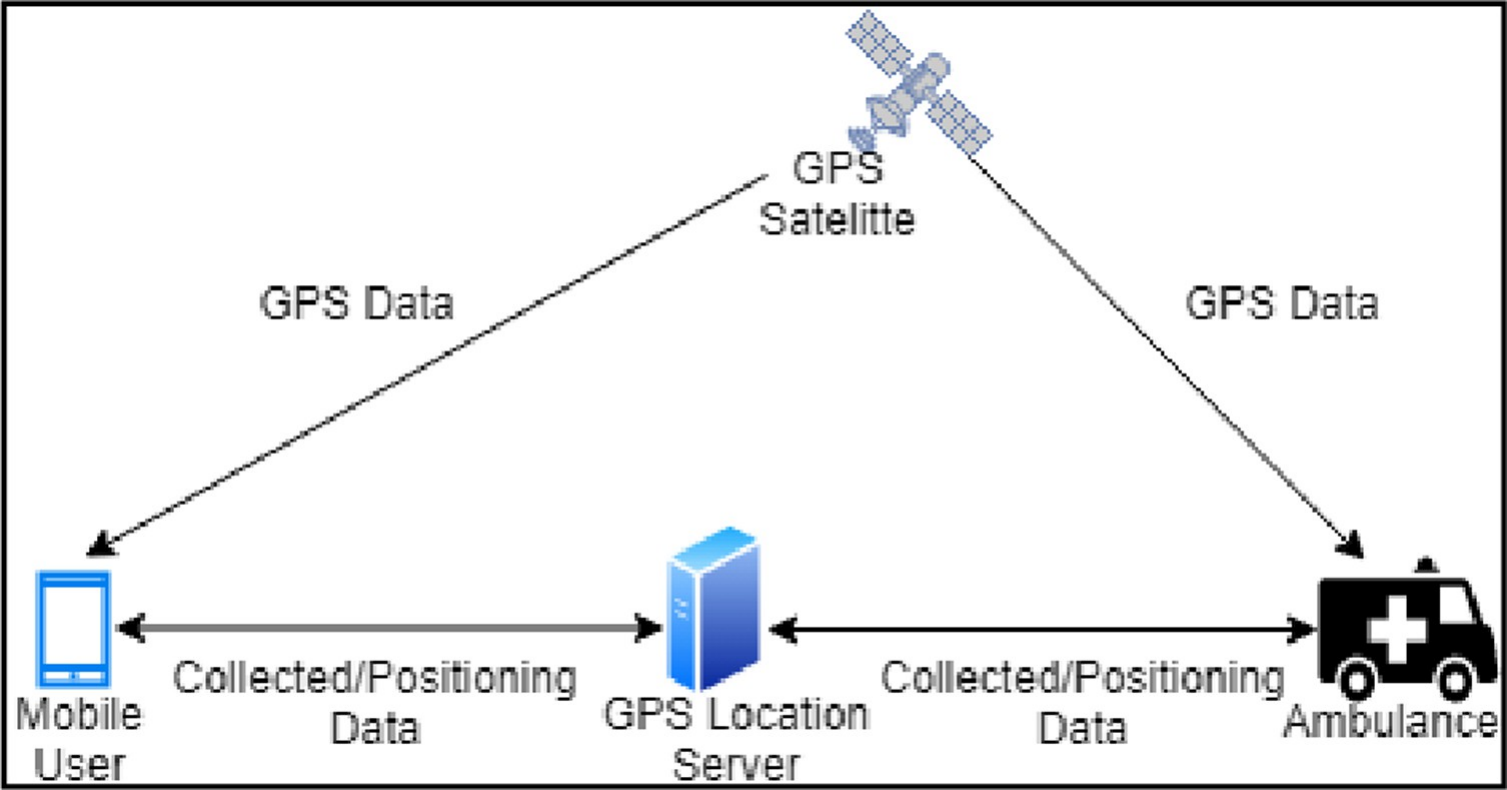
GPS system architecture.

Fig 1 represents the working of the GPS service to trace an EV and update its location in real-time on the GPS server. Navigation of the mobile devices by smartphone only works in areas served by reliable cellular service, which requires considerable financial resources. General packet radio services (GPRS) may provide imprecise directions due to changes in road names, traffic deviations due to road repair, and the emergence of new roads [2]. Updates of GPRS may reduce the chances of incorrect directions, but doing so imposes a financial burden. The GPRS satellite signal is weakened as it traverses through the atmosphere, impacted by the reflections, from objects such as tall buildings or large rock surfaces, along its route to reach a vehicle. Hence, the signal’s travel time increases, resulting in inaccurate location information in vehicular ad-hoc networks [3].

**Fig 2:**
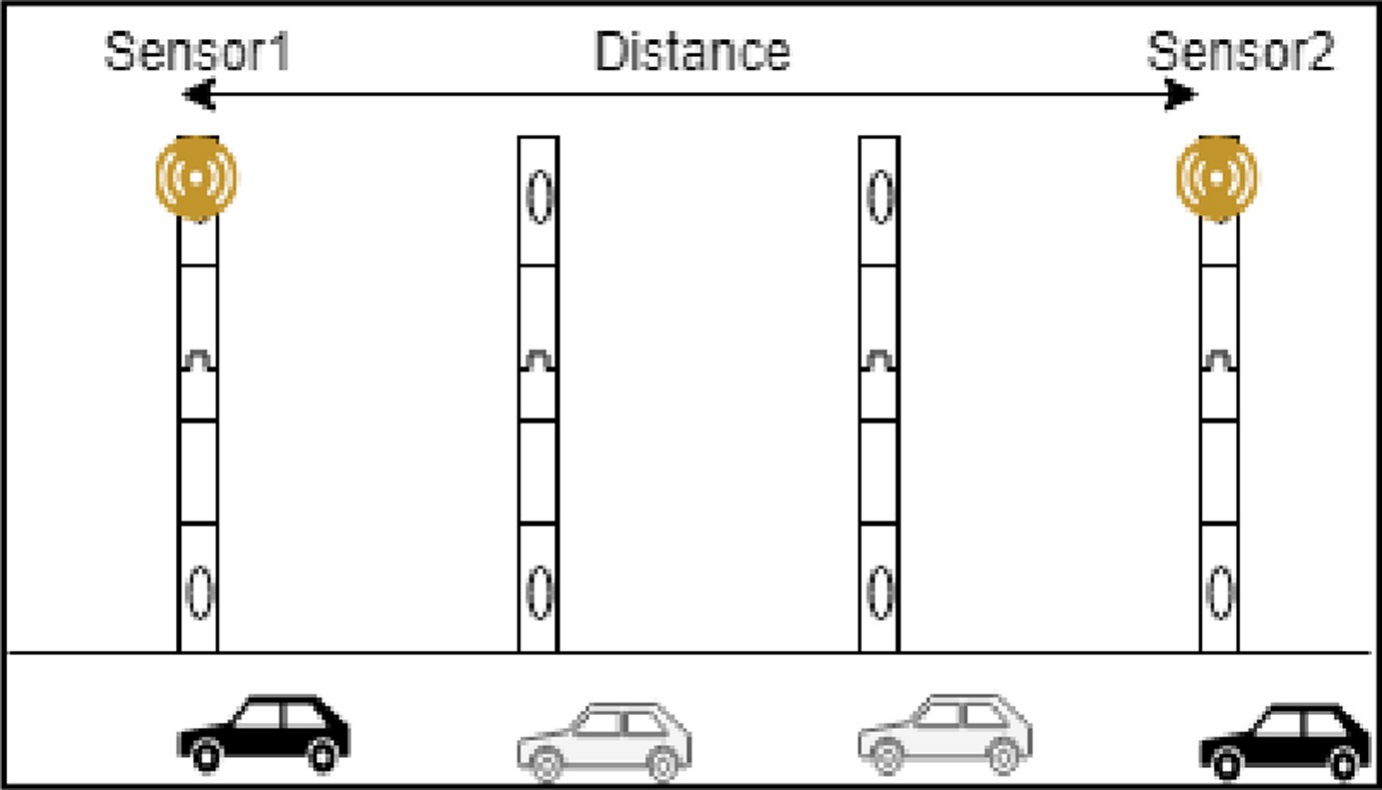
Sensor-based vehicle monitoring.

Environmental temperature change and extreme air turbulence affect the performance of sensors set to monitor vehicles, as presented in the Fig 2. Active sensors such as lasers, light detection and ranging, or millimetre-wave radars gauge the distance of an object by measurement of the travel time of a signal emitted by sensors and reflected by the monitored object [4]. The low spatial resolution, slow scanning speed, and interference between active sensors of the same type pose a big problem of less accurate location information. Hence, it necessitates an intelligent transportation system that provides efficiency in cost and performance. Accordingly, an increase in traffic congestion levels in the urban areas appears to be a challenging task and adversely impacts a country’s economy.

The conventional techniques employed for moving EVs, such as GPS, GPRS and sensor-based methods, are primarily based on mobile IPv4 (MIPv4) technology. These methods are impaired by frequent network handoffs, resulting in more message communication due to the triangle routing of MIPv4 and service interruptions. To address this issue, the present paper proposes a unique approach for automatic lane clearance for an EV, using mobile IPv6 (MIPv6), the next generation internet protocol and wireless networks [5, 6] to communicate the information an approaching EV to nearby vehicles. The key benefit of MIPv6 is adapted such that even though the EVs change their locations and IP addresses, the pre-existing connections are maintained.

The proffered approach comprises two main IPv6 based network connections: wired and wireless. Vehicles in the proximity of an EV get notified proactively, which helps in providing the lane clearance accordingly. In addition, the information of the approaching EV is communicated to a nearby roadside unit (RSU) that sends the received information to other vehicles and RSUs. Therefore, notifies the vehicles that are beyond the range of an EV. Thus, on receiving the alert message, vehicles that obstruct the clear path of the EV move over sideways. The concept can also be used for other real-world applications such as traffic regulations, detection of a police car or fire-fighting vehicle sirens.

The rest of the paper is organized as follows: Section 2 discusses previous works related to this problem domain; Section 3 includes a detailed description of the proposed method. Verification and performance evaluation of the proposed design are presented in section 4. Finally, closing remarks and conclusions are noted in section 5.

## 2. Related works

The volume of vehicular traffic is symptomatic of the level of growth and development in the modern-day world. The number of transport vehicles around the globe has been persistently increasing, and the capacity of the roads cannot cope with traffic; therefore, it creates hours-long traffic jams. The GPS sensors deployed in smartphones help collect a huge amount of location data, and smartphone use is rapidly increasing in locating and managing the stored information [7]. But, smartphones cannot measure traffic activity data directly. Alternatively, traffic activity can be captured by cameras or inductive loops incorporated in streets and traffic lights; however, it is very expensive to deploy camera sensors in large numbers [8].

The floating car data method locates the vehicle’s bearings via mobile phones or GPS to collect real-time traffic data [8–10]. Mobile phones or GPS act as sensors in this method and collect data pertinent to the vehicle’s location, speed, and moving direction. The collected data are sent anonymously to a central unit that extracts useful information and disseminates it to drivers on the road. A commonly adopted approach for detecting vehicles is active sensors [11] that determine the distance by measuring the elapsed time between the arrivals of the reflected signal previously emitted by the GPS sensors. However, active sensors are affected by low scanning speed and low spatial resolution. Optical sensors were deployed to provide visual information for traffic sign recognition, lane detection, or more, without any modifications to road infrastructures [12]. Optical sensors that acquire data in a non-intrusive way fall under passive sensors. Passive sensors are less expensive in comparison to active sensors. But the detection of the vehicle in the case of the passive sensors is more challenging because of the varying shape, size, illumination, and background of the materials around the environment [13].

The shortest path algorithm was used for the automatic EV rescue system [14]. The server in this rescue system maintains a database of the EV and its current status. If any accidental emergency happens, the main server communicates the location via GPS. Subsequently, the server checks the EVs that are available and free to select the one that is nearest to the accident site, using the Dijkstra algorithm. This system is limited by its dependency on a GSM message, a queue-based technique that suffers from latency. Traffic flow management was discussed in [15] to provide a smooth pathway for EVs based on RFID technology. The microcontroller also detects a stolen vehicle, and communicates that information to the control room via GPS service.

LiveViewGPS’s Live Trac project [https://live.liveviewgps.com] provides several benefits: directing an EV via the quickest routes, avoiding traffic situations and accidents, and determining the vehicle location to find the emergency team reachability [16]. The project also helps maintain the check on activities of emergency medical services. The RFID and GPS avoid unnecessary traffic congestion between emergency and non-emergency cases [17]. This system does not require any human intervention and is fully machine-controlled. This method needs details regarding the initial point and the endpoint of the travel of the mobility device; therefore, it may not work properly if any information is missing or ambiguous.

The communication of the approaching EV can provide a green channel throughout and help EVs avoid waiting at traffic signal junctions [18]. On reception of an alert message, the traffic signal changes from red to green to let the EV through and reverts to red once the EV has crossed the junction. Implementation of the routing protocol can be effective and dynamic for inter-vehicle communications. Although various methods such as video cameras, GPS, and infra-red sensors are available, inductive loops are the simplest method for detection of the vehicle [19].

**Fig 3:**
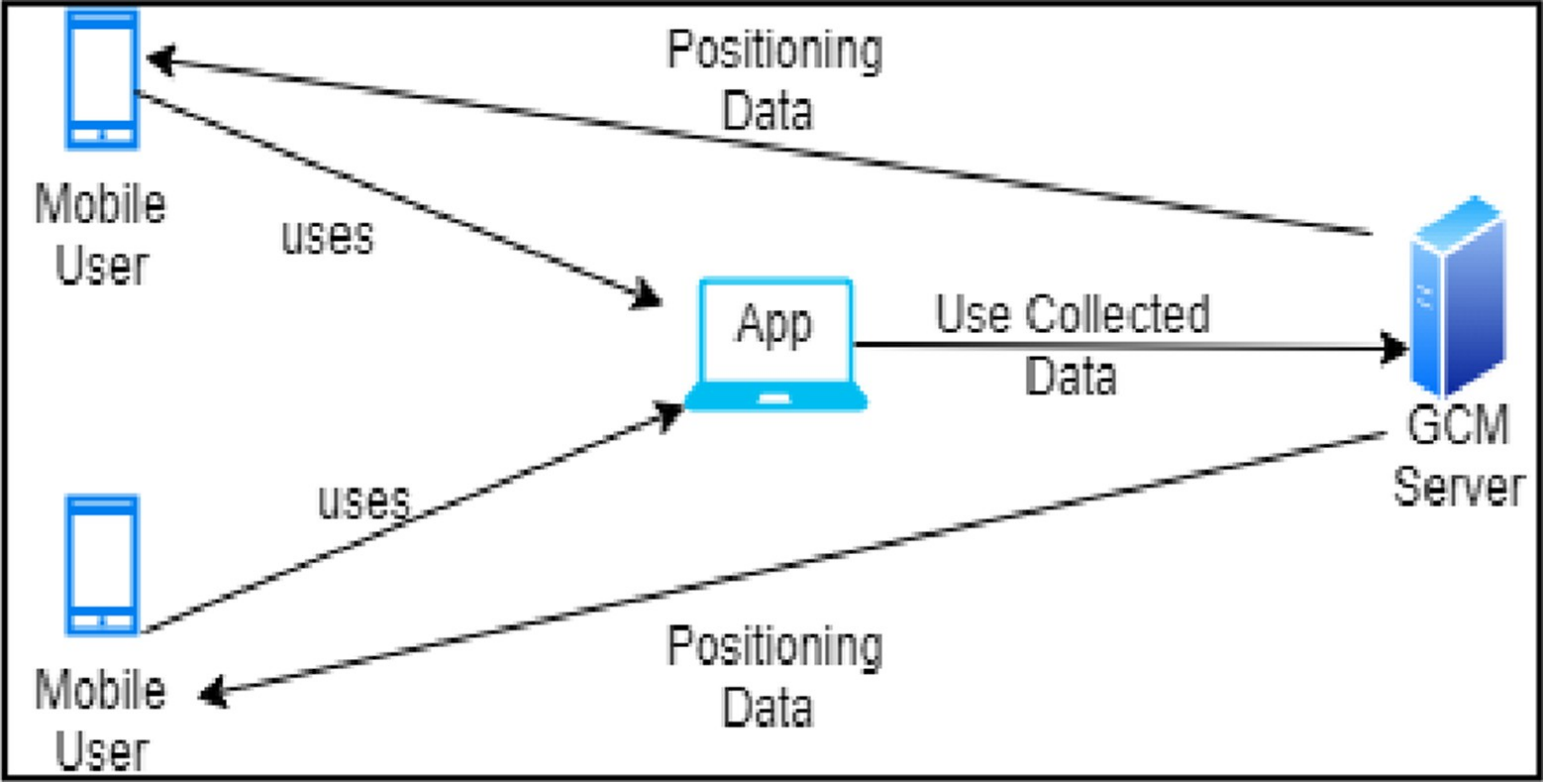
Android-based application.

An android-based application that can be used to track an EV (ambulance to the hospital–one of the EV), based on google cloud messaging (GCM) and GPS, to derive details of the hospital, was reported in [20]. The hospital’s application receives the messages from the users with the android-based application via the GCM server and responds accordingly, as shown in Fig 3. The application also helps the user track the EV to its exact location and determine the estimated time of travel of the EV to the emergency spot. Consumption of network bandwidth and battery power is minimized in this scheme due to the usage of the GCM [21]. Table 1 summarizes the existing lane clearance methods and their pros and cons.

**Table 1:** Comparison of lane clearance methods.

Method	Merits	Demerits
Cameras or inductive loops	Easy installation	Very expensive to deploy these sensors in large numbers
Floating car data	Collects the real-time traffic data	Centralized approach
Active sensors	Determines the distance parameter by measuring the time taken for the reflected signal	Low scanning speed and low spatial resolution
Passive sensors	Less expensive	More challenging because of the varying shape, size, illumination and background
RFID + GPS	Avoids unnecessary traffic congestion; fully machine-controlled	It may not work properly if any information is missing or ambiguous.

The diversity of vehicle range, lane discipline, and high population density of the vehicles are the major challenges in the deployment of intelligent transport systems. Moreover, huge installation and maintenance costs are required to fix the sensors [22]. Therefore, there is a need for a mechanism that can use the existing road infrastructure and provide the required facilities at a minimum cost.

## 3. Proposed approach

The proposed approach employs the concepts of geographic addressing and IPv6 communication [23] for road infrastructure. The extended address space, ease of configuration, embedded security and enhanced mobility support make IPv6 the most suitable technique for vehicular communications [24]. The assumption in the proposed approach for connecting RSUs is based on the existing underground cabling. Fiber optic cables have significantly more signal carrying capacity than metallic and coaxial cables. The former provides faster transmission because it is less susceptible to noise. Optical fiber is a backbone for broadband networks in most global infrastructures for the internet technique. Fiber-optic cables are laid down across the state and national highways and government-owned lands. Underground cabling also serves other purposes, such as electric power transmission, distribution, and telephone wiring. The extant underground cabling along the roads and pathways can be used to establish the connection between RSUs.

**Fig 4:**
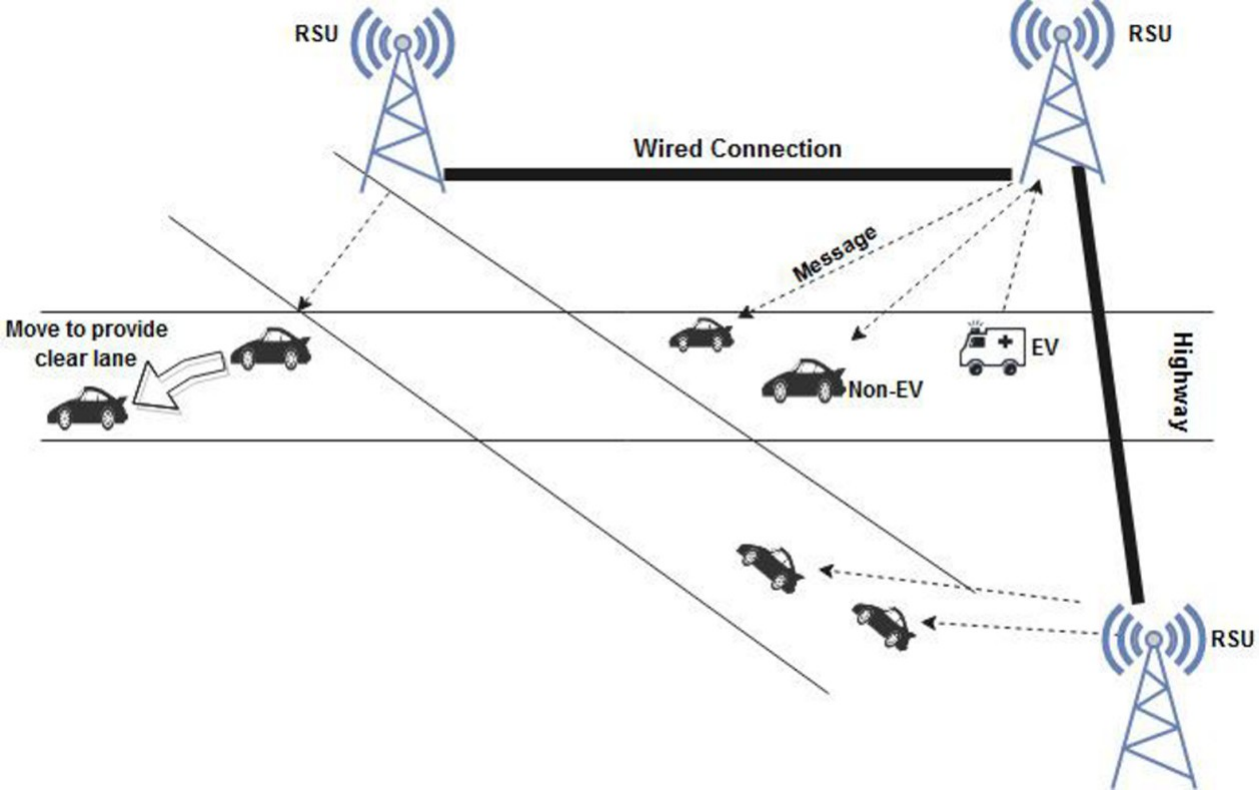
Communication over a MIPv6 based network.

The proposed approach comprises MIPv6-based wireless network connections; EV to non-EV, EV to RSU, and RSU to non-EV. As shown in Fig 4, the EV sends a message to the nearby vehicles and RSU. The RSU shares the received information with the other vehicles and RSUs to provide a clear lane to the EV. The proposed concept can also be deployed with other applications/services such as traffic regulations, detection of a police car or fire-fighting vehicle.

**Fig 5:**
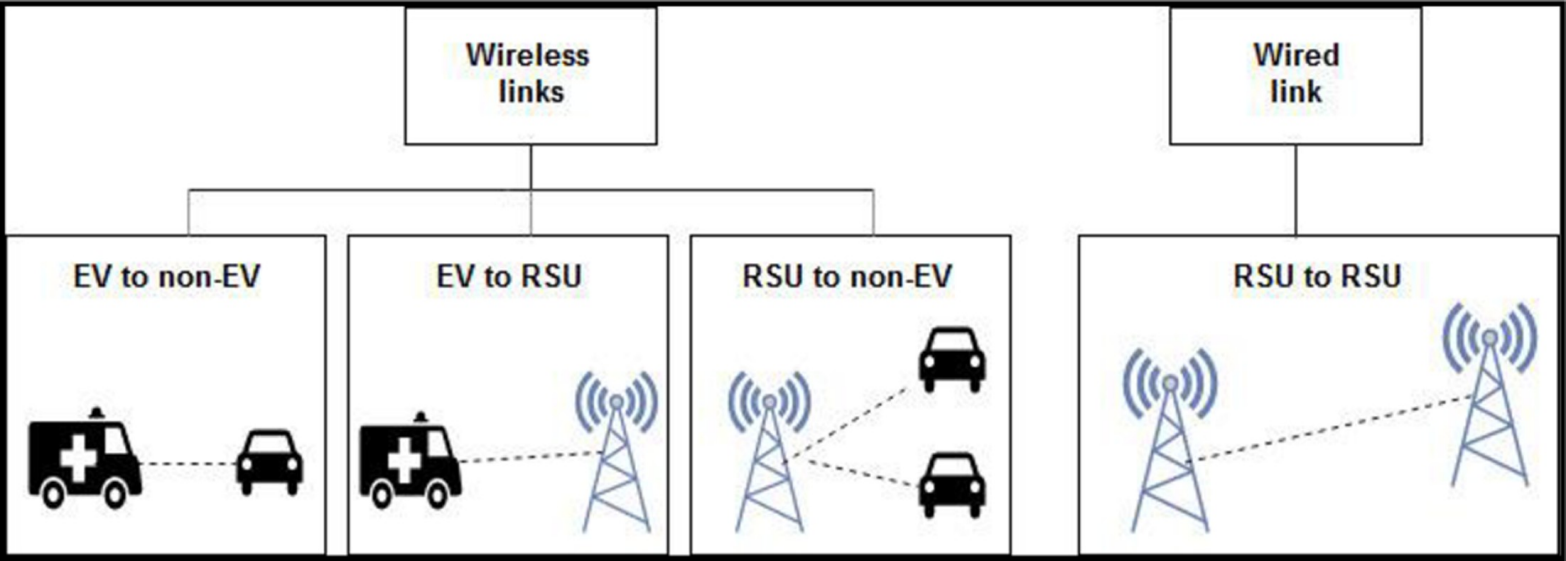
Communication links in the proposed approach.

The communications links for the proposed approach are depicted in Fig 5. Here, there are three wireless links: EV to non-EV, EV to RSU and RSU to non-EV. Furthermore, there is one wired connection link that is among the RSUs. RSU to non-EV uses a wireless communication link, and it assures the notification of the approaching EV to the non-EVs that are not within the range of the EV.

**Fig 6:**
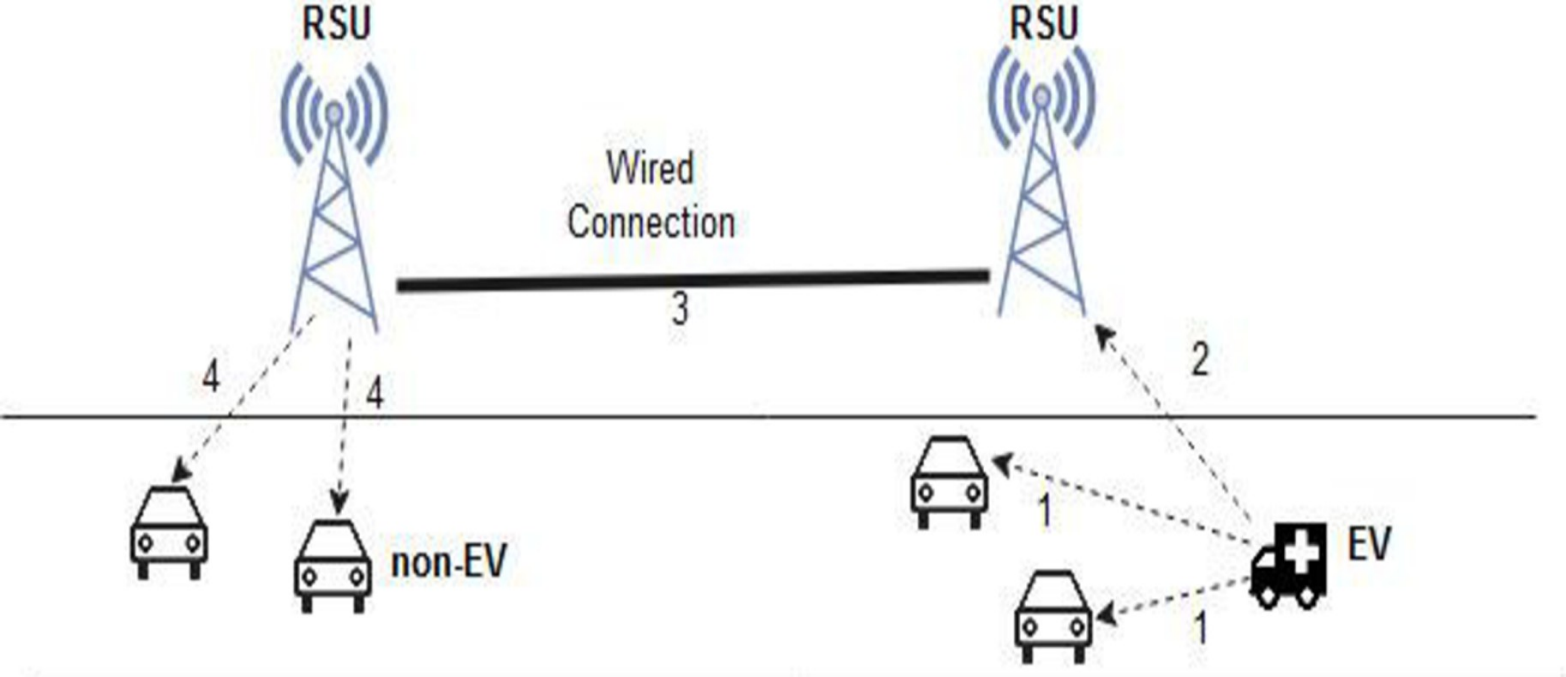
Message flow of the proposed approach. 1 and 2 –Direct message communication from EV to other non-Evs and the router (RSU) within its range. 3—Wired communication among the routers (RSUs). 4—Broadcasting of the message from the router (RSU) to the non-Evs.

Fig 6. represents the message flow of the proposed approach. Here, the numbers 3 and 4 denote information passing of the approaching EV to the vehicles that are not within the range of the EV and the RSU. Table 2 defines the notations used in the proposed approach.

**Table 2:** Notations used.

Notation	Description
*S*	Source address
*D*	Destination address
*CoA*	Care-of address
*Sbit*	Signaling bit pattern: 0 –Direct, 1 –Indirect
*Msg*	Message
*HoA*	Home-of address

The intricacies of the message communication of the proposed approach are as follows,

- EV to non-EV: When an EV moves from one location to another, it requires a clear lane to reach the intended destination on time to provide the requested service. It sends the following message update to the non-EVs within its range. Initially, the value of *Sbit* is 0.
  ***EV → non-EV:*** *S*_*EV*_, *D*_*non-EV*_, *CoA*_*EV*_, *Sbit*, *Msg*_*EV_non-EV*_
  The EV sends the notification message update to the nearby non-EVs, to indicate its presence on the lane. On receiving the notification, the non-EVs move over to leave the clear lane available to the approaching EV.
- EV to RSU-1: The EV also sends a message to the RSU. The RSU can broadcast that an EV is on the route to the other RSUs and the vehicles that cannot receive the information directly from the EV. The value of *Sbit* is 0 since the direct link is established from EV to non-EV.
  ***EV → RSU-1:*** *S*_*EV*_, *D*_*RSU-1*_, *CoA*_*EV*_, *Sbit*, *Msg*_*EV_RSU-1*_
- RSU-1 to RSU-2: A RSU can send updates to the other RSUs, via a wired connection to make the information available beyond the signal range of the EV. It helps in a better coverage area. Moreover, existing deployed underground cabling can be used in the wired connection mode, leading to a lower installation cost.
  ***RSU-1 → RSU-2:*** *S*_*RSU-1*_, *D*_*RSU-2*_, *HoA*_*RSU-1*_, *HoA*_*RSU-2*_, *Msg*_*RSU-1_ RSU-2*_
- RSU-2 to non-EV: resume RSU sends the received information to the vehicles. It helps when vehicles cannot detect the information of the approaching EV directly. Therefore, *Sbit* is 1 in this case.
  ***RSU-2 → non-EV:*** *S*_*RSU-2*_, *D*_*non-EV*_, *HoA*_*RSU-2*_, *Sbit*, *Msg*_*RSU-2_ non-EV*_

Therefore, the vehicles far from the approaching EV can get the update beforehand and remain organized to provide a clear way.

## 4. Prototype implementation and performance evaluation

### 4.1 Experimental setup

**Fig 7:**
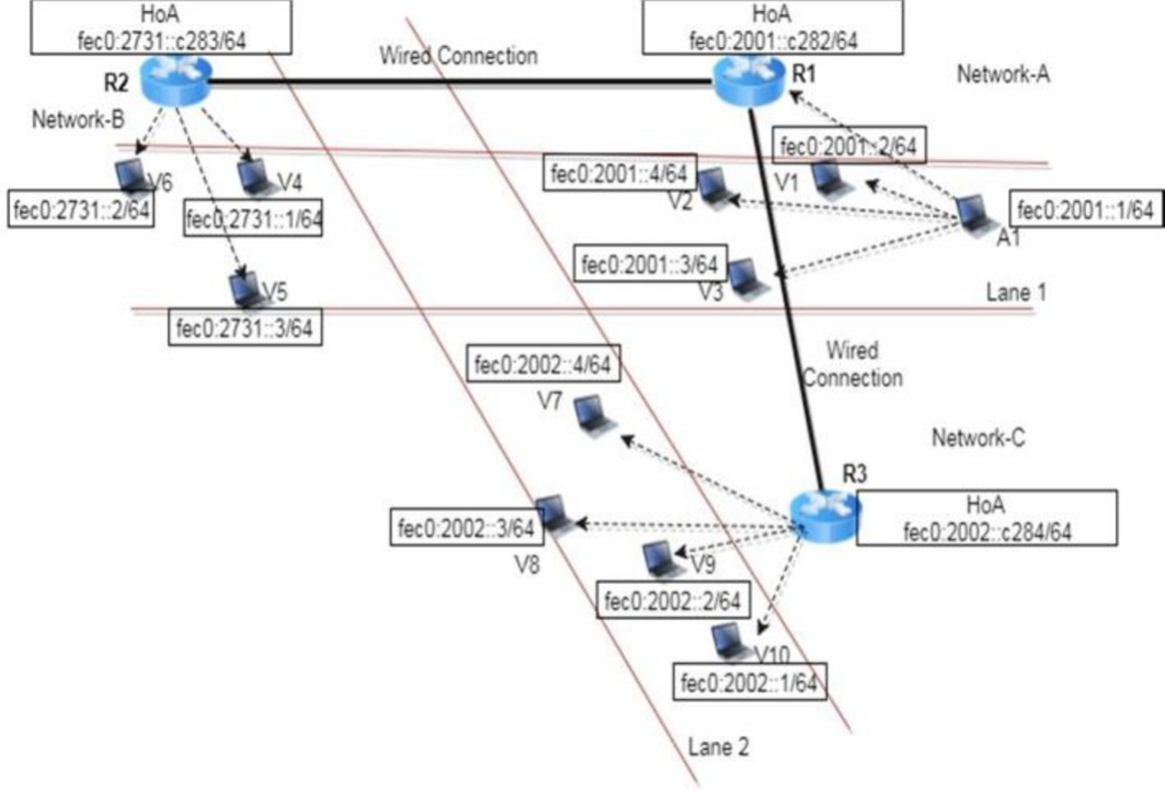
An experimental prototype design of the proposed approach.

The proposed approach was verified empirically by the implementation of a prototype. In the proposed prototype, a router was used as an RSU that integrates a 4-port switch and wireless access point. The router is compatible with virtually all the major operating systems, and supports the IPv6 protocol. The design of the prototype was constituted of three networks: network-A, network-B, and network-C, which depicts the MIPv6 mechanism. Each network deploys a router that maintains its respective home-of address (HoA) with IPv6 format and is connected via the wired connection, as shown in Fig 7. The network-A consists of four mobile nodes (MNs), in which A1 depicts the approaching EV and V1, V2, and V3 typify other vehicles in lane 1 (Fig 7). Router R1 broadcasts a router advertisement (RA) message; A1 and the vehicles under the R1 range receive the RA message as the vehicles move from the other network to network-A. Subsequently, they attain a new care-of-address (CoA) by the stateless address autoconfiguration (SLAAC) method. In network-B, three MNs depict vehicles (V4, V5, and V6) and router R2. R2 broadcasts the RA and follows the SLAAC; therefore, a new CoA gets assigned to the vehicles V4, V5, and V6. Moreover, the connection among the MNs is wireless and follows the IPv6 packet format. Similarly, network-C follows the SLAAC method for the vehicles—V7, V8, V9, and V10. Network-A illustrates the direct communication link from the EV to the vehicles nearby and the router. The network-B and network-C demonstrate the working of the vehicles in the lane that is not within the range of the approaching emergency vehicle A1.

Further subsection discusses the performance evaluation of the proposed approach. The parameters such as signaling overhead, message transmission time, packet delivery rate, propagation delay, end-to-end delay, and handover delay have been considered for the performance evaluation. Table 3 represents the symbols used in the performance evaluation.

**Table 3:** Symbols used in the performance evaluation.

Symbol	Description
CN	Correspondent node
BU	Binding update
LU	Location update
B_W_, B_WL_	Bandwidth of the wired and wireless links
T_W,_ T_WL_	Message transmission time in wired and wireless links
S_Msg_	Packet size for the notification message
T_IP_	Time for the IP configuration
T_sig_	Delay due to signaling
A_r_	Packet arrival rate at the router
S_r_	Rate at which router can process the packets
T_MV_	Exchanging the router solicitation (RS) and advertisement (RA) messages
C_LU_, C_BU_, L_PD_	Cost of location update, binding update, packet delivery
N_MN_, N_CN_	Number of the vehicle, router and emergency vehicle
D_MN_R_, D_MN_CN_	Distance between MN and router & MN and CN
T_res_	Stability time of the MN in the network
C_tran_BU_, C_tran_LU_	Transmission cost involved in the LU and BU signaling
C_proc_	Processing cost in the LU procedure
S_ar_	Session arrival rate

### 4.2 Message transmission time

The total time from the beginning to the end of the transmission of a message over a communication link is the message transmission time [25]. In the proposed approach, the EV broadcasts notification messages to the vehicles nearby, and the routers over a wireless link. It is the direct communication and reports the presence of the approaching EV. The time for the notification message to reach the nearby vehicles and the router can be calculated as follows,

$$T_{WL}=\left(\frac{S_{Msg}}{B^{WL}}\right)$$ (1)

Furthermore, the vehicles beyond the approaching EV’s direct range receive indirect notifications via routers, such as R2, as shown in Fig 7. The time taken to transmit the message from one router to another is as follows,

$$T_{W}=\left(\frac{S_{Msg}}{B^{W}}\right)$$ (2)

**Fig 8:**
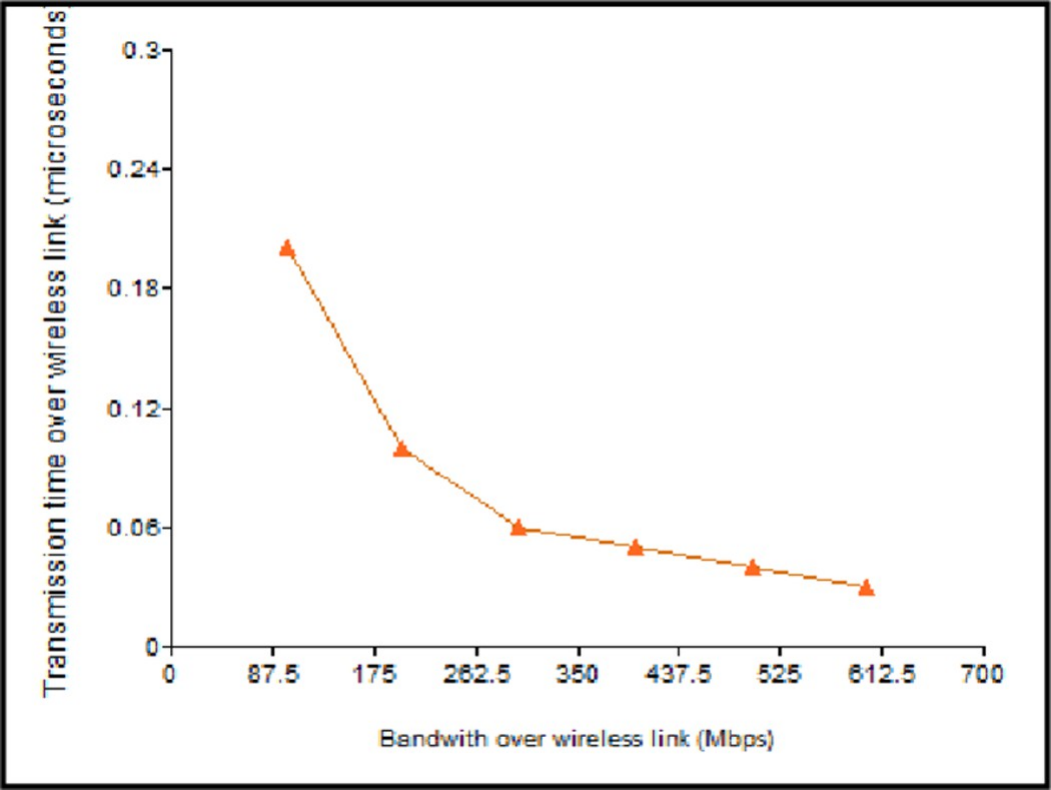
Message transmission time vs bandwidth over the wireless link.

As shown in Fig 8, the transmission time reduces with the increase in the bandwidth. However, with the rise in the value of the *B*_*WL*_, *T*_*WL*_ tends to be very less, resulting in the discarding of packets at the destination end. Therefore, the TP-Link router with 300 Mbps is used in the proposed approach that provides the optimal value for T_WL_. If *B*_*WL*_ is less, then the packet consumes more time to reach the destination node, and if it is more, it results in packet loss.

**Fig 9:**
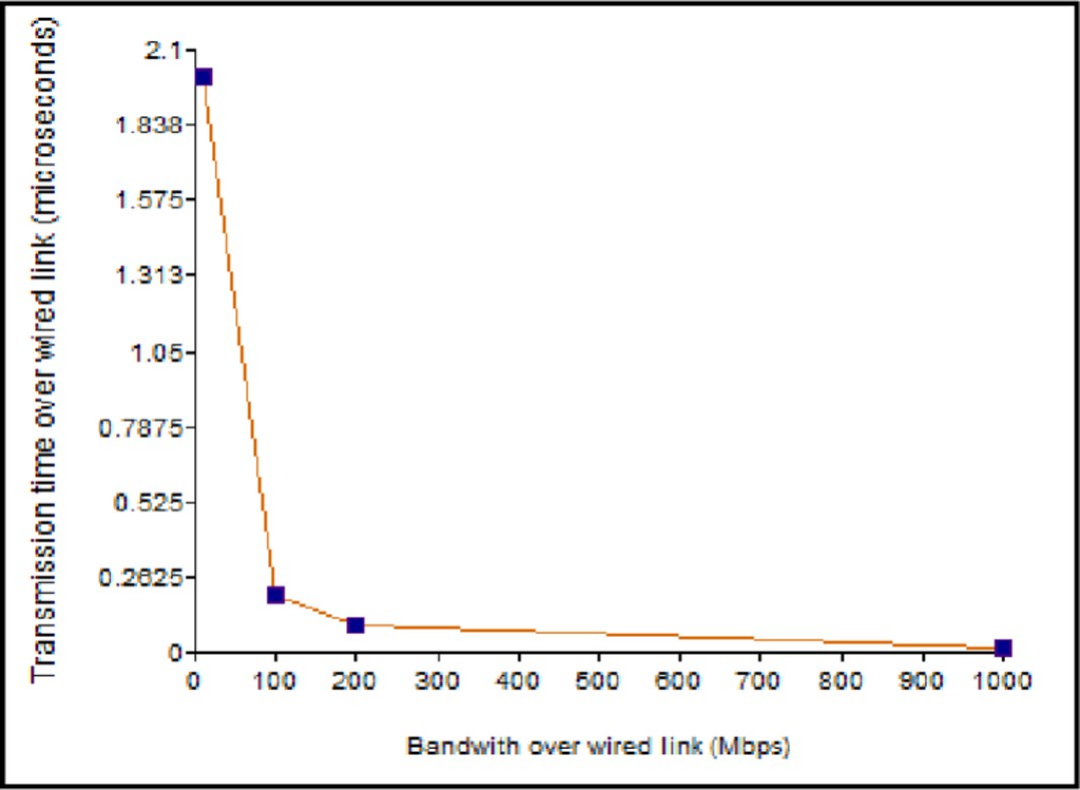
Message transmission time vs bandwidth over the wired link.

Underground fiber optic cabling is used for the wired connection between the routers. As the *B*_*W*_ value increases, the transmission time for a packet reduces, as shown in Fig 9. The wired connection follows the same behaviour as the wireless connection but has an extremely lower transmission time for a given bandwidth. As a result, the notification message of the approaching EV can reach other vehicles outside the range limits in a short duration.

### 4.3 End-to-end delay

The time taken by a data packet transmitted from a designated source to the specified destination node is defined as the end-to-end delay. The latter is dependent on the physical medium and the congestion level of the network. The end-to-end delay is the summation of the transmission delay (*d*_*trn*_), propagation delay (*d*_*pro*_), processing delay (*d*_*prc*_) and queuing delay (*d*_*que*_) as expressed below,

$$End-to-end\;delay=d_{trn}+d_{pro}+d_{prc}+d_{que}$$ (3)

Transmission delay *d*_*trn*_ can be computed for the respective medium. Here, the impact of the physical medium of the links on the propagation speed is given in the wired and wireless links are 2 x 10^8^ meter/second and 3 x 10^8^ meter/second, respectively.

The wired connection between the routers can be measured to determine the value of the distance parameter, and the propagation speed value can be taken from Table 3. As a result, the propagation delay in the wired connection can be determined by using the distance-speed formula as shown in Eq 4,

$$d_{pro}=\left(\frac{Distance\;between\;the\;routers}{Propagation\;speed\;in\;wired\;link}\right)$$ (4)

The packet communication session can involve an EV to a router, an EV to nearby vehicles, or a router to the vehicles within the router range. Thus, propagation delay in the wireless connection, as per the respective communication session, can be calculated as given in Eq 5,

$$d_{pro}=\left(\frac{Distance\;between\;the\;nodes}{Propagation\;speed\;in\;wireless\;link}\right)$$ (5)

When a router receives the data packet from the approaching EV or another router in the network, it processes the packet. The processing delay is dependent on the error bit and the next routing destination. The methodology proposed in this study addresses the value of *Sbit* in the notification message that determines if the packet is received directly or indirectly. No additional processing occurs at the router; thus, *d*_*prc*_ tends to a null value. The message received by a router is transmitted to the other router and the vehicles that are beyond the direct range of the approaching EV. The router places the message packets in the queue if the arrival rate of the packets is faster than the transmission rate of the router. Hence, the time duration, from the time of reception of a packet by the router until it is transmitted further, is *d*_*que*_, and it is represented as shown in Eq 6,

$$d_{que}=\left(\frac{1}{Sr-Ar}\right)$$ (6)

Also, S_r_ is given by,

$$S_{r}=\left(\frac{d_{trn}}{average\;S_{Msg}}\right)$$ (7)

In the proffered approach, when it receives the notification message packet, the router broadcasts the packet to the other routers and the vehicles in the vicinity. Hence, *d*_*que*_ is equivalent to the negligible. From Eqs (1), (2), (4), (5) and (6), the end-to-end delay is expressed as follows,

$$E2E\;delay=d_{trn}+d_{pro}$$ (8)

**Fig 10:**
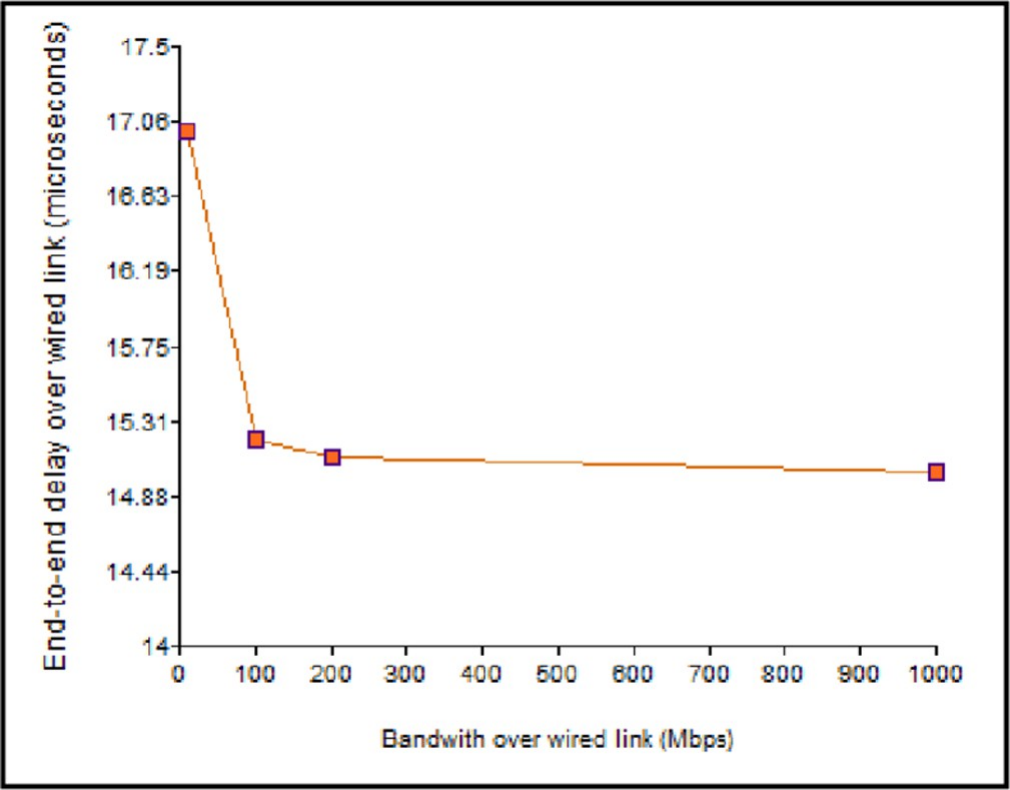
Impact of bandwidth on the end-to-end delay (wired link).

**Fig 11:**
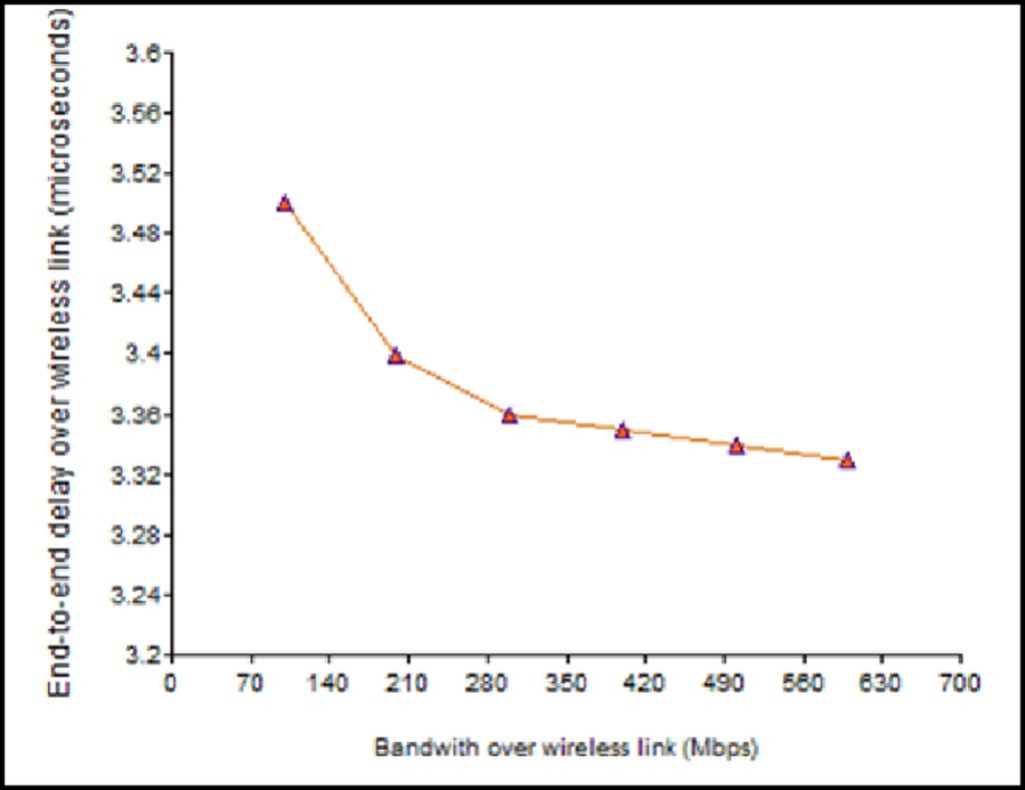
Impact of the bandwidth on the end-to-end delay (wireless link).

When *B*_*W*_ is increased, the transmission delay is less. The value of *d*_*pro*_ was estimated as 15 microseconds, per Eq (4). As a result, Fig 10 illustrates that the end-to-end latency falls with the increase in *B*_*W*_. Correspondingly, *d*_*pro*_ was computed as 3.3 microseconds using Eq (5) in the wireless connection. The delay reduces with the increase in the *B*_*WL*,_ as shown in Fig 11.

The integrated (RFID + GPS) approach has a less end-to-end delay in providing the service than the android-based application because it directly deals with the change of the posted traffic signal from an approaching EV. In an android-based application, the delay may happen due to the GPS service’s incorrect location update or slow signaling. Furthermore, the shortest path algorithm uses the Dijkstra algorithm to determine the shortest path from an accident spot to the nearby hospital. Thus, EV takes less time to reach the hospital than the other approaches.

In the approach delineated in the investigation, vehicles and the router within the range of the EV’s sirens receive the direct notification message. Furthermore, vehicles that are not within the EV’s siren range get the updates from the router and move out of the pathway to provide a clear lane to the EV. Therefore, the proposed approach takes less time as it does not depend on server updating and information processing units.

### 4.4 Packet delivery ratio (PDR)

The ratio of the number of data packets successfully delivered to the number of packets sent computes the PDR [26]. PDR depends on the notification message broadcast by the approaching EV, and the reception of the message by the router and the nearby vehicles. In the case of the indirect notification, The PDR is computed as,

$$Packet\;deliver\;ratio=\left(\frac{Packets\;received}{Packets\;Sent}\right)$$ (9)

**Fig 12:**
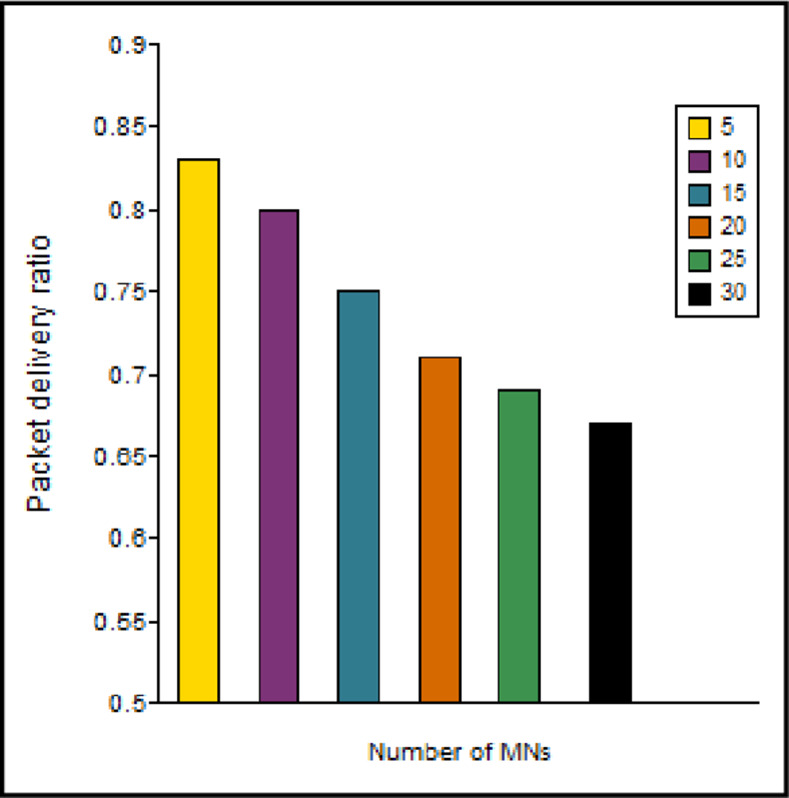
PDR vs number of the MNs.

As shown in Fig 12, PDR becomes poorer as the MNs increase in number because an increase in the count of the MNs leads to more packet collisions and signaling overhead.

### 4.5 Handover delay

The handover delay is one of the vital processes in mobile networks [27]. The exchange of RA and RS takes place between the router and the MN whenever an MN moves to a new network. The proposed approach initiates the detection of the MN, and adds to the *T*_*MV*_ until the process of IP configuration begins. Therefore, the respective latency is denoted as shown in Eq 10,

$$T_{MV}=RA+RS$$ (10)

$$T_{IP}=Delay\;for\;IP\;configuration+Delay\;for\;forwarding\;table\;updation$$ (11)

Next, MN sends the BU to the router that revised the association of the HoA and CoA parameters of the MN. Subsequently, the router sends the binding acknowledgement (BA) response to the MN. These exchanges of the binding messages are represented as,

$$T_{sig}=BU+BA$$ (12)

Based on the values of Eqs (10), (11) and (12), the total handover delay can be estimated as,

$$Handover\;delay=T_{MV}+T_{IP}+T_{sig}$$ (13)

**Fig 13:**
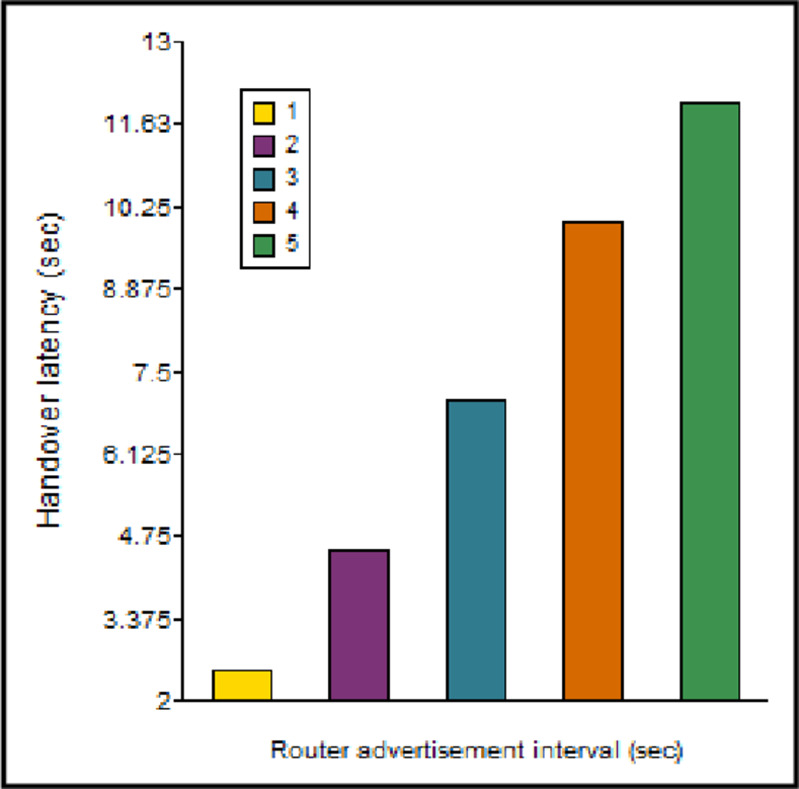
Handover latency vs. RA interval.

An increase in the RA interval increases the handover latency because T_MV_ is directly proportional to RA and RS, as may be seen from Eq (10). Fig 13 describes the behaviour of the handover delay concerning the interval of the RA. If the value of the RA interval is extremely low, the handover delay would be less, but the signaling overhead is more. On the contrary, if the RA interval is high, it increases the handover delay and is inefficient for real-time applications. Thus, an optimal value of RA interval is selected that balances the handover delay and the signaling overhead in the network.

The combined (RFID + GPS) approach consumes less time than the shortest path algorithm, as it is immune from any storage overhead on the database server. However, it generates more delay in sharing the information. The comparisons of GPRS and an IPv6-based network without an internet connection are displayed in Table 4. The proposed approach does not use server-side processing and GPS services; hence, it faces the least delay and consumes less time than other information-sharing methods. Thus, the proffered scheme performed with a better response time than the other approaches.

**Table 4:** Comparison of GPRS and IPv6 based network.

Attribute	GPRS	Next generation MIP
Connectivity	Loss of connectivity during roaming	Sustains connectivity
Response time between entities	Uplink and downlink	Less (auto configuration + prefix node)
Delay	Incurs with queuing delay	Less (nearest neighbour discovery)
Bandwidth consumption	Works on various GSM bands	Less in IPv6 network-based communication
Cost factor	The charge depends on the volume of data	Depends on the network
Dependency on infrastructure / equivalent	Database (home location registers and visitor location register)	No dependency on database
Impact on emergency aspect and power	Not efficient when an entity enters a roaming area (disconnects)	It can work in real-time applications and has low battery consumption

### 4.6 Signaling cost

The proposed approach depends on the notification messaging from an EV to the routers and nearby vehicles. The handover procedure includes the LU and the BU of the vehicles and EV in the network. Therefore, signaling cost considers the signaling overhead related to the LU and BU and the packet delivery measures [28]. Whenever any MN moves to a new network, RA and RS exchange the messages between the MN and the router. Furthermore, MN acquires the new temporary CoA as the LU happens. There can be several MNs in the network; therefore, N_MN_ is multiplied by the *C*_*LU*_ of each MN to determine the total cost, in the case of the LU. Hence, the cost related to the LU signaling can be given as shown in Eq 14,

$$C_{LU}=N_{MN}*\left(\frac{(2*D_{MN\_R}*C_{trans\_LU})+C_{proc}}{T_{res}}\right)$$ (14)

The cost related to the location update is inversely proportional to when MN stays in the network itself. If the MN stays for a comparatively lesser time, it is highly mobile, leading to high LU signaling cost; whereas, if it resides for the longest time, then signaling cost is less. Once LU takes place, MN sends the BU to the CNs to update the new location. Furthermore, CN updates the information and sends the BA to the MN. The binding update cost can be computed from Eq 15:

$$C_{BU}=\left(\frac{N_{MN}*N_{CN}*2*D_{MN\_CN}*C_{trans\_BU}}{T_{res}}\right)$$ (15)

The notification message exchange among them follows the association between the MN and the CN. The delivery of the data packets depends on the session arrival rate of each MN in the network, which is shown in Eq 16,

$$S_{ar}=\left(\frac{1}{E(T_{s1})+E(T_{s2})}\right)$$ (16)

where *T*_*s1*_ = time, for which MN is on the current network itself. *T*_*s2*_ = Time interval, when the MN moves from the current network to the next network. *E(T*_*s1*_*)* can be deduced using the probability density function for the continuous variable, time. The integration over time is used, instead of the summation, because of the continuous distribution of the time interval.


$$E(T_{s1})=\int f(T_{s1})dT_{s1}$$ (17)

$$\text{E}({\text{T}}_{\text{s}2})=\int \text{f}({\text{T}}_{\text{s}2}){\text{dT}}_{\text{s}2}$$ (18)

Similarly, the packet delivery cost is proportional to the overhead entailed in the lookup of the location database of the MNs, at the router end. The location database maintains the association between MNs and CNs. Thus, the packet delivery cost is,

$$C_{PD}=N_{MN}*N_{CN}*S_{ar}*Database\;lookup\;cost$$ (19)

Therefore, the signaling cost can be derived by using (14), (15) and (16),

$$Signaling\;cost=C_{LU}+C_{BU}+C_{PD}$$ (20)

**Fig 14:**
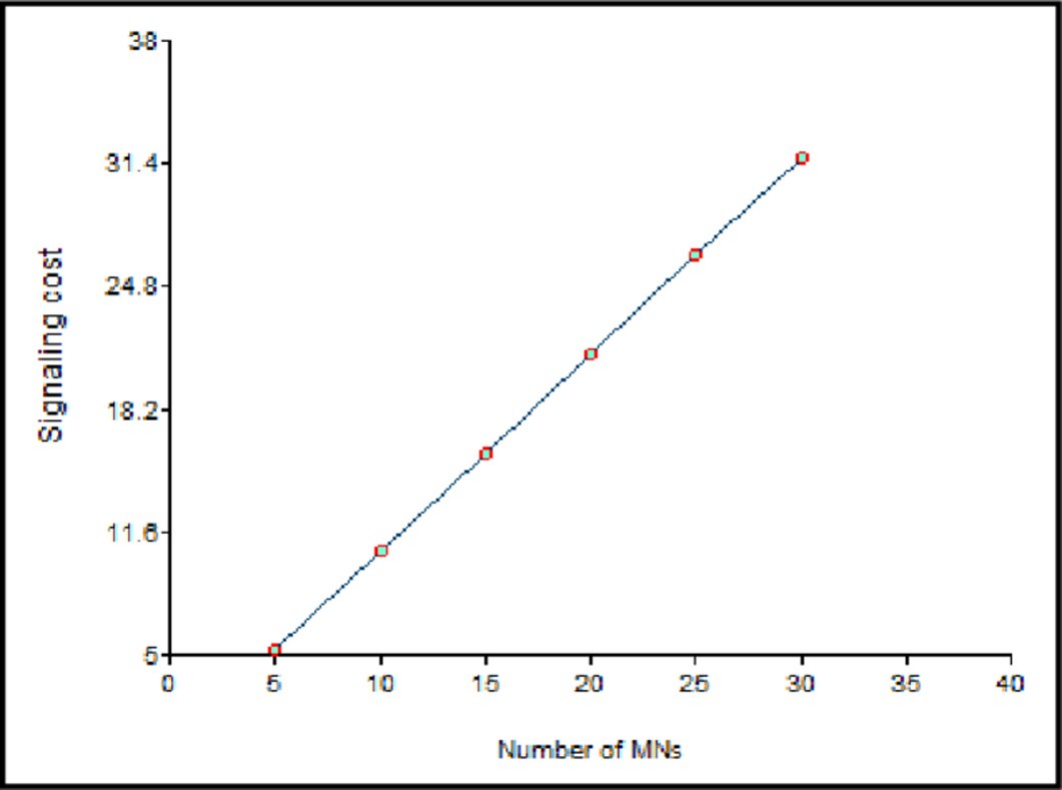
Signaling cost vs. the number of MNs.

The impact of the count of the MNs, on the signaling cost, is given in Fig 14 for the respective values of *C*_*trans_LU*_, *C*_*proc*_, *N*_*CN*_, *C*_*trans_BU*_, *S*_*ar*_, database lookup cost at router and *T*_*res*_. *C*_*LU*_, *C*_*BU*_, and *C*_*PD*_ are computed using Eqs (14), (15) and (19). Subsequently, the total signaling cost can be calculated by summing up the binding, location update, and packet delivery costs. It may be quite apparent that signaling cost increases with the number of MNs. The count of vehicles (non-EV) has been on a monotonic rise among advanced and developing countries. Consequently, the overhead increases due to the binding updates.

**Fig 15:**
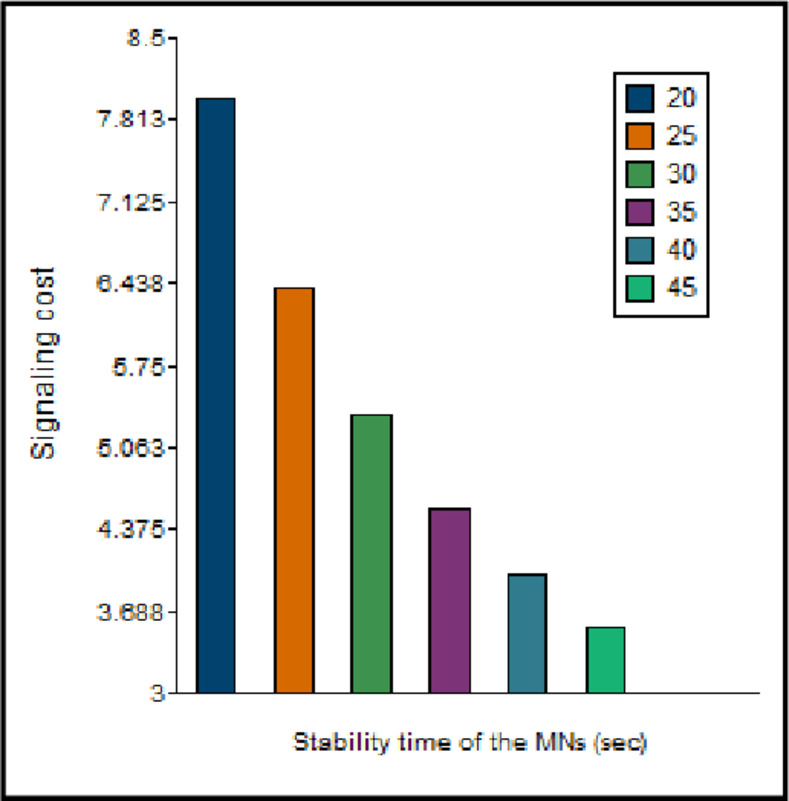
Signaling cost vs. stability of MNs.

The duration of MN within a network affects the signaling cost [29]. The behaviour of the signaling cost concerning the *T*_*res*_, considering *N*_*MN*_ *= 5* and *N*_*CN*_ *= 1*, is shown in Fig 15. As *T*_*res*_ increases, the signaling cost for LU and BU decreases. Hence, the overall signaling cost is lower when the MNs are not highly mobile within the network.

The shortest path algorithm faces more signaling overhead than the unified (RFID + GPS) approach because the server controls the tracking of the EV and the traffic lights, ensuing in additional sharing of update messages. The communication is only between the EV and the traffic signals on the RFID + GPS approach [30]. The signaling overhead of android-based applications is less than the other discussed methods. It focuses on the communication between the user and the hospital pertinent to the android application. The latter resorts to GCM service for free messaging and GPS service to get updates on the current location. Independent of radiofrequency and GPS signaling mechanisms, the proposed approach incurs the least signaling overhead.

### 4.7 System failure

There is no high impact due to the failure of any of the pre-mentioned routers in the network. The EV can directly notify the nearby vehicles; hence, moving forward to the next network, where the router can receive the notification from the EV further to notify the farthest vehicles in the clear lane. Moreover, there is a wired connection among the routers. It provides faster transmission and less interference. If there is any issue related to the underground cabling connecting the routers, the connection may get dropped, but the rest of the network remains unaffected. There can be other sources of interference, such as wireless speakers and Bluetooth devices. The property of dual band in the routers can minimize the interference by changing the channels accordingly.

## 5. Conclusion

The loss of life due to transit delays in the arrival of the EV service can be prevented by providing a clear lane to the EV. Conventional methods lean on sensors, GPS and GCM servers to accomplish the intelligent transportation system. The use of sensors has limitations, such as low scanning speed and spatial resolution. The server-based methods have overhead storage issues, and the latency concomitant impairs GPS-based methods with location updates. Hence, a new approach for lane clearance based on the MIPv6 protocol was discussed in this paper. The delineated method uses extant underground wiring for the connection between routers that minimize the expenses incurred in the deployment process, and provides faster transmission of data packets. The impact of the number of moving vehicles (MNs) on the packet delivery ratio was reviewed by numerical analysis. The observed results show that the proposed approach significantly alleviates the end-to-end and handover latencies. This approach can also be used in other real-world applications associated with logistic optimization.

## Supporting information

S1 Data (XLSX)
